# Increased Virulence and Large-Scale Reduction in Genome Size of Tetraploid *Candida albicans* Evolved in Nematode Hosts

**DOI:** 10.3389/ffunb.2022.903135

**Published:** 2022-06-27

**Authors:** Amanda C. Smith, Hassan Rizvi, Meleah A. Hickman, Levi T. Morran

**Affiliations:** ^1^ PhD Program in Genetics and Molecular Biologist, Emory University, Atlanta, GA, United States; ^2^ ORISE Fellow, Oak Ridge Institute for Science and Education (ORISE), Oak Ridge, TN, United States; ^3^ Department of Biology, Emory University, Atlanta, GA, United States

**Keywords:** *Candida albicans*, *Caenorhabditis elegans*, fungal genomics, experimental evolution, ploidy, innate immunity

## Abstract

*Candida albicans* is an opportunistic fungal pathogen of humans, yet the within-host dynamics of *C. albicans* infection are not clear. While *C. albicans* is commonly diploid, it exhibits a range of ploidies, including tetraploidy. Previous work found that tetraploid *C. albicans* populations exhibited rapid adaptation and significant genome instability when evolved *in vitro*. Host immune function alters the rate and magnitude of *C. albicans* virulence evolution, but the effects of the host immunity on tetraploid *C. albicans* populations are unclear. Here, we tested the effects of the host immunity on genome stability and virulence evolution of tetraploid *C. albicans* using experimental evolution. We selected for *C. albicans* increased virulence within either immunocompetent or immunocompromised *Caenorhabditis elegans* hosts. After nine passages we observed a response to selection for increased virulence. Both populations exposed to either immunocompetent or immunocompromised hosts increased virulence after passage through *C. elegans* hosts. However, the *C. albicans* populations passaged through immunocompetent hosts under selection exhibited unique temporal dynamics, a rapid increase in virulence and then subsequent loss of virulence. Most *C. albicans* populations exhibited genome size reduction within six passages, however populations exposed to immunocompetent hosts exhibited the most rapid transition to ~diploid. Therefore, we found that tetraploids rapidly increase in virulence and decrease genome size within host environments. Further, the combination of selection for greater virulence in the presence of immunocompetent hosts results in major virulence fluctuations and genome size changes. Thus, host immunity significantly impacts the evolutionary trajectories of tetraploid *C. albicans*.

## Introduction

*Candida albicans* is an opportunistic fungal pathogen of the human microbiota (Nash et al., 2017). While mostly commensal, it does cause both benign mucosal infections as well as deadly bloodstream infections (Pfaller and Diekema, 2007). These infections are more common in immunocompromised individuals (Pfaller and Diekema, 2007). *C. albicans* often maintains a stable relationship with the host, but the host contains multiple stressors, including the immune system, that exert selective pressures on the microbe. Beyond exerting selective pressure, the host immune system induces genome instability in *C. albicans* (Smith et al., 2021). *C. albi*c*ans* is normally a diploid organism, but it has a highly labile genome that rapidly undergoes large-scale genomic changes including loss-of-heterozygosity, aneuploidy and whole ploidy shifts in presence of physiological stressors (Forche et al., 2011), including the host environment (Forche et al., 2009; Ene et al., 2018; Forche et al., 2018; Smith and Hickman, 2020). This combination of host-induced effects, selection pressure and genomic instability, may significantly alter the evolutionary trajectory of *C. albicans* populations. Yet, little is known regarding the impact of host generated genetic variation on the long-term evolution of *C. albicans*.

Genetic variation is needed for evolution by natural selection. The rates at which beneficial mutations arise or novel genotypes are generated in a population is critical for determining how quickly adaptation occurs (Fisher, 1930). However, the way in which this genetic variation is generated varies across species. While meiosis and sexual reproduction are common, *C. albicans* does not reproduce sexually and must rely on other means of generating genetic variation to facilitate adaptation to environmental changes. *C. albicans* undergoes a process known as the parasexual cycle, where it is normally diploid but exists on a spectrum from haploid to highly polyploid (Suzuki et al., 1982). These various ploidy states have been isolated from patients as well as laboratory settings. The reduction from tetraploid to diploid is stochastic and can generate a reassortment of alleles, loss of heterozygosity (LOH), and even transient aneuploidy (Bennett and Johnson, 2003; Forche et al., 2008; Hickman et al., 2015). This ability to rapidly undergo substantial genomic changes may allow for *C. albicans* populations to rapidly adapt in the presence of different stressors, including the host immune system.

Ploidy is known to impact the rate of adaptation. Indeed, tetraploid populations of *Saccharomyces cerevisiae* adapt faster than both diploids and haploids to different environmental stressors *in vitro* (Selmecki et al., 2015). Therefore, tetraploid populations may rapidly adapt to host environments as well. Our previous results demonstrated that tetraploid *C. albicans* are more unstable than diploids within the host (Smith and Hickman, 2020), and that a functional host immune system increases *C. albicans* genome instability and facilitates rapid adaptation (Smith et al., 2021). Yet, the impact of host immunity on the rate of adaptation of tetraploid *C. albicans* has not been investigated.

Here, we use a *C. elegans* host model to investigate how the host environment impacts the evolutionary trajectories of tetraploid *C. albicans* populations under selection for increased virulence. We used experimental evolution to select for virulence while passaging tetraploid *C. albicans* through either immunocompetent or immunocompromised *C. elegans.* We predicted virulence evolution would occur faster in immunocompetent hosts due to the selective pressure and genome instability imposed by the host immune system. We found that tetraploids rapidly became more virulent in immunocompetent hosts, but then exhibited reductions in virulence following several additional passages. Further, virulence evolution also occurred under selection in immunocompromised hosts, albeit at a slower rate compared to *C. albicans* in immunocompetent hosts. After nine passages through hosts, regardless of immune status*, C. albicans* populations exhibited increased virulence and reductions in genome size.

## Methods

### Strains and Maintenance

We used *C. albicans* strain MH128 (RBY18) (Bennett and Johnson, 2003) and two *C. elegans* strains *glp-4* (bn2) and AU37 (*glp-4* (bn2); *sek-1* (km-4). Both *C. elegans* strains have a mutation in glp-4 which leads to temperature sensitive sterility at 15°C (Rastogi et al., 2015), allowing for population control during experimental evolution. AU37 has an additional mutation in sek-1, which leads to enhanced susceptibility of pathogens (Kim et al., 2002). Yeast strains were stored at -80°C and maintained on YPD (yeast peptone dextrose; 1% yeast extract, 2% bactopeptone, 2% glucose, 0.004% adenine, 0.008% uridine) at 30°C. Strains were initially struck onto YPD agar plates from frozen glycerol stocks and incubated at 30°C for 48 h and single colonies used as the “parental strain” in subsequent *in vivo* experiments. Nematode populations were maintained at 20°C on plates containing nematode growth media (NGM) with *E. coli* (*Escherichia coli*, OP50) for a food source. *C. elegans* were transferred to a new plate containing freshly seeded *E. coli* every 3-4 d. For experimental evolution, plates were seeded with both *C. albicans* and *E. coli* and supplemented with 0.2 g/L streptomycin to inhibit overgrowth of *E. coli*.

### Host Preparation and Seeding

NGM plates are seeded with a mixture of *E. coli* and *C. albicans* 24 h prior to host preparation. To seed plates, *C. albicans* cultures were inoculated into 3 mL YPD and incubated overnight at 30°C. Cultures were adjusted with ddH_2_O to a final concentration of 3.0 OD_600_ per mL (1 OD_600_ is approximately 3 x 10^-7^ CFU/mL). Simultaneously, a single colony of *E. coli* was inoculated into 50 mL LB and incubated for 24-48 h at 30°C. The *E. coli* culture was pelleted and washed twice with 1 mL ddH_2_O. The pellet was weighed and diluted to final concentration of 200 mg/mL. For experimental evolution experiments, *C. albicans* treatment plates had 1.25 µL of *C. albicans* and 6.25 µL of *E. coli* and were brought to a final volume of 50 µL. The entire 50 µL was spotted onto the center of a 35-mm-diameter NGM plus streptomycin agar plate, followed by incubation at room temperature overnight before the addition of eggs or transferred nematodes. Synchronization of nematode populations were performed similar to (Feistel et al., 2019; Feistel et al., 2020; Smith and Hickman, 2020; Smith et al., 2021).

### Experimental Evolution

Six replicate populations of 50 C*. elegans glp-4* (bn2) and AU37 (*glp-4* (bn2); *sek-1* (km-4)) were infected with a mixture of *C. albicans* and OP50. We measured virulence as host mortality, and imposed selection for increased virulence by passaging *C. albicans* from only the first 50% (25 worms) of the population that died. *C. elegans* populations were counted daily and transferred to new plates with the same seed culture every 48 h. Dead nematodes were collected daily by picking into in .5 mL M9 buffer (3 g KH_2_PO_4_, 6 g Na_2_HPO_4_, 5 g NaCl, 1 mL of 1 M MgSO_4_ in 1 L of H_2_O) until 50% of the population died. Following 50% death, the nematode carcasses were centrifuged for 30 s at max speed. The supernatant was removed and 500 µL of 3% bleach was added to the pellet to kill any bacteria or yeast on the surface of the nematodes. After two min of bleach treatment, the tube was centrifuged for 30 s at max speed. The supernatant was removed and 500 uL of M9 was added to the pellet and centrifuged for 30 s at max speed. This step was repeated two additional times. The worm pellet was then exposed to manual disruption with a motorized pestle. After 1 min of manual disruption, all the intestinal extracts were inoculated into 2 mL of YPD + 0.034mg/L chloramphenicol to prevent any bacterial growth. After 24 h of growth at 30°C, extracted *C. albicans* was used to seed for the subsequent generation with fresh OP50. New, synchronized populations of *C. elegans* were used for each passage. To ensure changes in virulence were due to host exposure, *C. albicans* with OP50 was passaged on NGM + streptomycin in the absence of hosts.

For the treatment under no selection, 25 dead and alive nematodes were randomly selected at the same time point as when 50% hosts died in the selection treatment and yeast was extracted and passaged as described above.

### Flow Cytometry

*C. albicans* extracted from *C. elegans* at the end of each passage were stored in glycerol stocks at -80°C. *C. albicans* from glycerol stocks were inoculated in YPD and incubated overnight at 30°C. Flow cytometry was performed similar to (Smith and Hickman, 2020). Briefly, following logarithmic growth, cells were fixed with 95% ethanol, treated with 50 μl of RNase A (1 mg/ml) for 1 h at 37°C with shaking, then 50 μl proteinase K (5 mg/ml) and incubated for 30 min at 37°C, and resuspended in 50 μl Sybr green (1:50 dilution with 50:50 TE; Lonza, catalog no. 12001-798, 10,000×). Samples were sonicated to disrupt any cell clumping and subsequently run on an LSRII flow cytometer. To calibrate the LSRII and serve as internal controls, the reference diploid (SC5314) and tetraploid (mating product of RBY16 & CHY477) (Bennett and Johnson, 2003) strains were used.

Flow cytometry data were analyzed using FlowJo, by plotting the fluorescein isothiocyanate A (FITC-A) signal against the cell count. Two peaks were observed, the first representing the G1 mean and the second peak representing the G2 mean, which has double the genome content of the G1 peak and therefore twice the fluorescence. Genome size values were calculated using the G1 mean and compared to standard diploid and tetraploid control strains.

### Statistical Analysis

To measure changes in *C. albicans* virulence, the average time to 50% death for each population was tested against the time to 50% death in the initial passage using a Kruskal-Wallis followed by *post-hoc* Dunn’s multiple comparisons test for all treatments and selection regimes using Prism V8 Software. To evaluate changes in mean time to host death relative to passage 4, we performed a Mann-Whitney U test comparing the mean time to host death in the initial passage, along with passages 4, 8, and 9.

To evaluate if there were differences in the time to approximately diploid, we performed a binomial generalized linear model (GLM) testing for the effects of host immune function, selection, and the interaction between immune function and selection using SAS (v. 9.4). Finally, to determine if changes in genome size correlated with changes in virulence, we performed a logistic regression analysis in SAS, testing for a correlation between the change in days until 50% death each passage (relative to the previous passage) versus the change in genome size for each passage (relative to the previous passage), for each experimental population. We performed separate analyses for the immunocompetent hosts and immunocompromised hosts.

## Results

### Host Immunity Coupled With Selection Facilitates Rapid Virulence Changes in *C. albicans*

**Figure 1:**
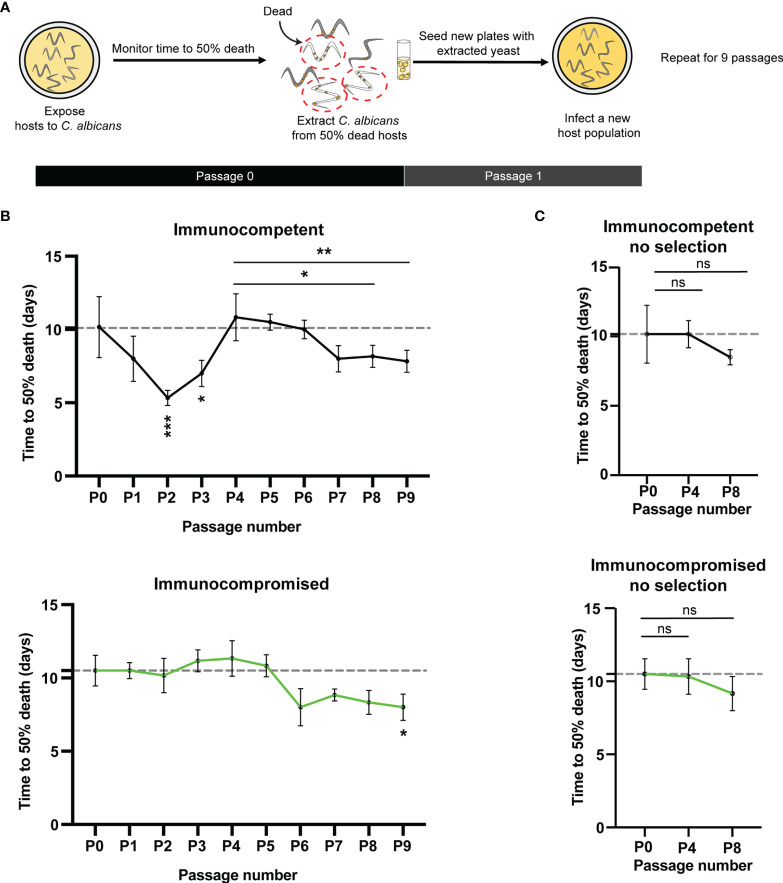
Tetraploid *C.albicans* evolve rapidly in immunocompetent hosts under selection **(A)** Experimental evolution schematic **(B)** top:Time to 50% death for ten passages in healthy, immunocompetent *(glp-4)* hosts. Plotted are the mean (n=6) and SD for each passage bottom: Time to 50% death for ten passages in immunocompromised (*glp-4; sek-1)* hosts. Plotted are the mean (n=6) and SD for each passage. Asterisks represent significant differences compared to the initial (PO) time point (***0.005, **p < 0.01, *p < 0.05 Kruskal-Wallis with post-hoc Dunn's multiple test). ns = not significant. Dashed line indicates the initial average time to 50% death. **(C)** top: Line graph representing the average and standard deviation for time to 50% death for immunocompetent *(glp-4)* osts not under selection for the initial passage (PO), passage 4 (P4) and passage 8 (P8). bottom: Line graph representing the average and standard deviation for time to 50% death for immunocompromised (*glp-4 sek-1)* hosts not under selection for the initial passage (PO), passage 4 (P4) and passage 8 (P8). Small dots represent individual data points. (ns, not significant, p > 0.05 Kruskal-Wallis with post-hoc Dunn’s multiple test).

Our goal was to determine the impact of tetraploidy on virulence evolution in a host environment. Therefore, we evolved tetraploid *C. albicans* in immunocompetent (*glp-4*) or immunocompromised (*glp-4; sek-1*) *C. elegans* hosts with and without selection for increased virulence for nine passages (**Figure 1A**). To impose a selective pressure, we passaged *C. albicans* from individuals that died most rapidly to the subsequent generation (**Figure 1A**). *C. albicans* populations passaged in both immunocompetent and immunocompromised hosts under selection evolved greater virulence during nine passages of experimental evolution (Fig. 1B). However, the rate and pattern of virulence changes differed considerably between treatments. Following passages 2 and 3, immunocompetent *C. elegans* infected with *C. albicans* exhibited significantly less time to 50% death compared to the initial passage, indicating increased *C. albicans* virulence (Fig. 1B, top, p = 0.0003 and p <0.043 respectively). Yet, by passage 4, immunocompetent host survival was again equivalent to hosts in the initial passage, with an average time to 50% death of 11 days (Fig. 1B, top). Despite the maintenance of the selective pressure, we observed no significant changes in survival for immunocompetent hosts in passages 5 and 6, relative to passage 4 (**Figure 1B**, top, p >.9999). Then, immunocompetent host survival time was significantly reduced in passages 8 & 9 relative to passage 4 (p < 0.013, p < 0.0065 respectively; Mann-Whitney U). Thus, *C. albicans* virulence increased again with continued selection after passage 4. C*. albicans* virulence in immunocompromised hosts under selection was relatively steady until we observed a decline in host survivorship in passage 6 (relative to passage 5) (**Figure 1B**, bottom, p = 0.0022). This change was then maintained throughout the remainder of the experiment, with passage 9 exhibiting greater virulence than the ancestor (**Figure 1B**, bottom, p = 0.0075). Overall, these results indicate that a functional immune system under selection facilitates rapid changes in *C. albicans* virulence, and alters the dynamics of virulence evolution in tetraploid populations of *C. albicans*.

To determine whether the changes in *C. albicans* virulence were a direct result of selection, we passaged *C. albicans* populations in the absence of selection for increased virulence. In the absence of selection, we observed that host immunity did not impact *C. albicans* evolutionary trajectories. Further, without selective pressure, we observed no significant changes in the average time to 50% death for either immunocompetent or immunocompromised hosts at passage four or eight, suggesting no substantial change in *C. albicans* virulence (**Figure 1C**). Therefore, host immunity alone, without imposed selective pressure did not drive changes to *C. albicans* virulence. Together, these data demonstrate that host immunity coupled with selective pressure can alter the evolutionary trajectories of tetraploid *C. albicans* populations.

### Selective Pressure and Host Immunity Determine Rate of *C. albicans* Genome Size Reduction

**Figure 2:**
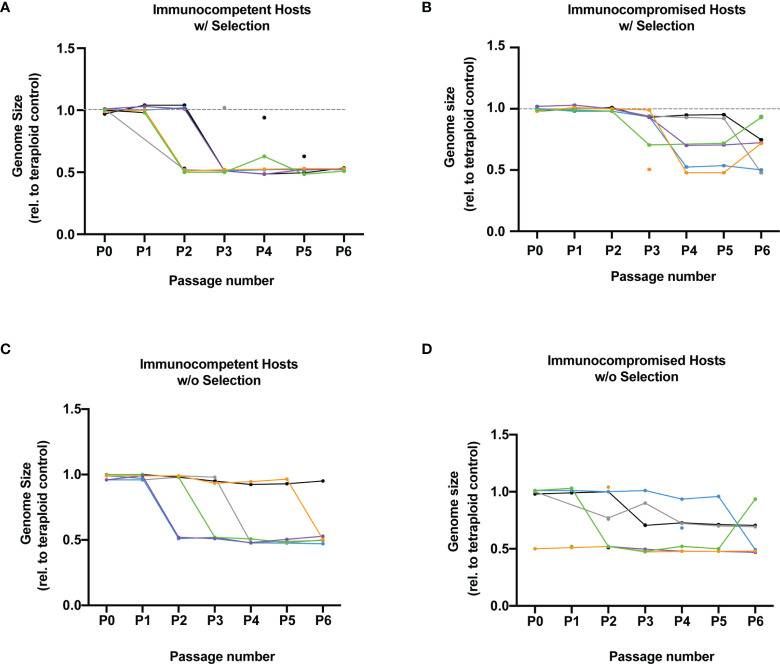
Tetraploids genome size change trajectories following evolution **(A)** Plotted is the genome size of *C. albicans* relative to a tetraploid control following passaging in immunocompetent hosts with selection for six passages. Each different colored line represents 1 of the 6 unique lineages. Multiple points indicate cell populations with multiple ploidies. **(B)** Same as **(A)** but for immunocompromised hosts under selective pressure **(C)** Same as **(A)** but for immunocompetent hosts not under selection **(D)** Same as **(A)** but for immunocompromised hosts not under selection.

We wanted to determine if selection and exposure to host immune function were also associated with changes in *C. albicans* genome size. We measured genome size with flow cytometry for each of the six replicate populations from each treatment (selection/no selection and immunocompetent/immunocompromised). We observed changes in genome size within 23 of our 24 populations throughout 6 passages of experimental evolution. Following six passages in immunocompetent hosts with and without selection, 11 out of 12 tetraploid populations had a reduction in genome size by approximately 50%, indicating diploidy (**Figures 2A, B**). In contrast, *C. albicans* passaged through immunocompromised hosts for six passages exhibited greater variability in genome size and significantly fewer transitions to diploidy (**Figures 2C, D**, p = 0.046), with 4 approximately diploid populations and 8 populations varying between tetraploidy and triploidy. This indicates that populations exposed to immunocompromised hosts transitioned to an intermediate ploidy state as opposed to rapidly transitioning to diploidy. Further, the absence of intermediate states within *C. albicans* populations exposed to immunocompetent hosts, suggests rapid concerted chromosome loss (CCL). While *C. albicans* exposed to immunocompromised hosts underwent a more gradual CCL from tetraploid to approximately diploid. Together, these results indicate that host immune function facilitates rapid genome size reduction from tetraploid to ~diploid in *C. albicans* populations. However, we did not detect an effect of selection on transitions to ~diploidy (p = 0.91). Further, changes in virulence were not statistically correlated with changes in genome size (immunocompetent p = 0.74, immunocompromised p = 0.12). Therefore, our data indicate that passage through immunocompetent hosts drove more rapid genome size shifts to ~diploid, however these changes in genome size were not directly correlated with changes in *C. albicans* virulence.

## Discussion

We tested the effects of selection for greater virulence and the host’s immune response on the evolutionary trajectories of tetraploid *C. albicans* populations. We found that selection within the host, regardless of immune status, can rapidly increase virulence (**Figure 1**). Additionally, passage through immunocompetent hosts resulted in more rapid transitions to ~diploid than passage through immunocompromised hosts, while passage through immunocompromised hosts led to greater levels of aneuploidy. Finally, selection for greater virulence within immunocompetent hosts produced different evolutionary dynamics than selection within immunocompromised hosts. Specifically, *C. albicans* populations passaged under selection within immunocompetent hosts evolved significantly greater virulence within two passages, lost virulence over the next two passages, and then subsequently exhibited a modest increase in virulence over the remainder of the experiment. Conversely, *C. albicans* passaged under selection within immunocompromised hosts exhibited a more subtle and gradual increase in virulence over the course of the experiment. Therefore, both the host immune response and the presence of selection determined the dynamics of virulence evolution within tetraploid populations of *C. albicans*.

*C. albicans* has a highly labile genome, which frequently carries aneuploid chromosomes following host exposure (Ene et al., 2018; Forche et al., 2018; Smith and Hickman, 2021), drug stress (Selmecki et al., 2006), and following the stochastic process of concerted chromosome loss in tetraploids (Bennett and Johnson, 2003; Berman and Hadany, 2012; Hickman et al., 2015). These higher ploidy states and genome instability help to facilitate evolution, with higher ploidy states undergoing evolution faster than diploids or haploids (Selmecki et al., 2015). We recently demonstrated that tetraploids are highly unstable in a host environment (Smith and Hickman, 2020) likely due to host immune stress (Smith et al., 2021). Here, we demonstrated that tetraploid *C. albicans* are highly unstable in a healthy host environment. Following only two passages in immunocompetent hosts under selection, all the replicate lines were approximately diploid or had mixed populations containing both tetraploids and diploids (**Figure 2**). This rapid transition to near-diploid happens very quickly and is unlikely due to the selective pressure. Rather, the rapid genome size reduction is likely the result of ploidy drive which posits *C. albicans* has a propensity to return to its baseline ploidy under nutrient limiting conditions (Gerstein et al., 2017). Indeed, populations exposed to immunocompetent hosts transitioned to ~diploidy at a faster rate than populations passaged through immunocompromised hosts. Interestingly, we also observed that many C*. albicans* populations dropped directly from tetraploid to near-diploid in immunocompetent hosts. Yet, we observed many intermediate near-triploid populations in immunocompromised hosts before reaching ~diploid. Transitions from tetraploidy to diploidy allow for different and novel combinations of chromosomes and allelic ratios which provide the raw material for populations to respond to selection. Aneuploidy is often a product of the parasexual cycle. In certain environments aneuploidy is beneficial. For example, in fluconazole *C. albicans* frequently gains an extra copy of the left arm of chromosome which it allows for greater resistance due to the extra copies of genes mediating drug resistance (Selmecki et al., 2006; Selmecki et al., 2008). However, aneuplodies often incur a fitness cost (Torres et al., 2007) and are often a transient state until baseline ploidy is reached (Yona et al., 2012). *C. albicans* exposed to both types of hosts likely underwent some transition to aneuploidy, yet *C. albicans* aneuploidy was not maintained in immunocompetent hosts. While changes in ploidy were not directly correlated with increased virulence, the dynamics of virulence evolution differed between *C. albicans* in populations of immunocompromised vs immunocompetent hosts, and changes in genome size may have driven these different evolutionary trajectories.

We observed a response to selection for increased virulence in our experimental populations, while our control populations did not significantly increase in virulence over the course of the experiment. Further, the dynamics of the response to selection differed depending on the host immune function. Specifically, we observed a significant increase in tetraploid *C. albicans* virulence exposed to immunocompetent hosts by passage two. However, the increased virulence exhibited in passage two within the immunocompetent hosts was not maintained. Conversely, selection within immunocompromised hosts did not result in a significant change in virulence until passage nine. We propose that virulence changes occurred faster in *C. albicans* under selection in immunocompetent hosts compared to immunocompromised hosts because the immune system may increase the strength of selection, in addition to inducing ploidy drive. In response to *C. albicans* infections, *C. elegans* produce antimicrobial peptides and reactive oxygen species (Hoeven et al., 2011; Pukkila-Worley et al., 2011; Dierking et al., 2016) to fight infection. We found that these immune stressors elevate *C. albicans* genome instability, which likely facilitated faster adaptation in immunocompetent compared to immunocompromised hosts. However, we observed a rapid loss of virulence which suggests that alleles contributing to greater virulence may have had deleterious pleiotropic effects with regards to either within host or *in vitro* growth. In the event that increased virulence was not associated with increased growth rates, either within the host or *in vitro*, then genotypes with greater virulence may have been outcompeted by genotypes that facilitated greater growth in either environment. Thus, a lack of correlation between virulence and growth rate may explain the “rebound” to lower virulence that we see in passage 2 in immunocompetent hosts under selection. Further, population bottlenecks resulting from host infection potentially decreased the efficacy of selection (Vega and Gore, 2017), which could have exacerbated the loss of rare alleles contributing to increased virulence. Such rapid changes in ploidy (**Figure 2**), are likely to produce many novel genotypes at low frequencies in populations. Overall, it is plausible that this loss of virulence in the immunocompetent hosts is a product of our passage scheme. Tetraploid *C. albicans* populations may more readily maintain virulence within infections of a single host or patient, which would limit serial bottlenecks and exposure to vastly different environments.

We found that tetraploids rapidly evolved virulence in just two passages in immunocompetent hosts under selection (**Figure 1B**). For comparison, diploid *C. albicans* evolved virulence in immunocompetent hosts by passage five (Smith et al., 2021). Together, this suggests that tetraploidy facilitates rapid adaptation, a result that is consistent with other recent evolution studies. For example, tetraploid *C. albicans* evolved faster than diploid *C. albicans* under antifungal drug selection (Avramovska et al., 2021) and tetraploid *Saccharomyces cerevisiae* adapted faster to raffinose selection compared to diploids (Selmecki et al., 2015). This accelerated adaptation is likely due to the higher mutation rates in tetraploids and frequent chromosome loss generating new favorable allelic ratios (Forche et al., 2008; Hickman et al., 2015).

Taken together our results suggest that the immune system generates large genomic changes that have significant phenotypic consequences. We demonstrated that under a selective pressure for virulence phenotypes, tetraploid *C. albicans* populations evolve rapidly within immunocompetent hosts. Thus, the host immune system may play a role in the shift of *C. albicans* from commensal to pathogen, and perhaps vice versa. Moreover, stressors such as the addition of antifungal drugs, which cause genome instability, whole-ploidy changes, and aneuploidy are also likely to impact the evolutionary dynamics of infection.

## Data Availability Statement

The raw data supporting the conclusions of this article will be made available by the authors, without undue reservation.

## Author Contributions

AS, LM, and MH designed the study. AS and HR conducted all of the experiments. AS and LM analyzed the data. AS, HR, MH, and LM wrote, reviewed and edited the manuscript. All authors contributed to the article and approved the submitted version.

## Funding

This research is supported by NSF DEB-1943415 (MH), NSF DEB-1750553 (LM), and Emory University startup funds (MH).

## Conflict of Interest

The authors declare that the research was conducted in the absence of any commercial or financial relationships that could be construed as a potential conflict of interest.

## Publisher’s Note

All claims expressed in this article are solely those of the authors and do not necessarily represent those of their affiliated organizations, or those of the publisher, the editors and the reviewers. Any product that may be evaluated in this article, or claim that may be made by its manufacturer, is not guaranteed or endorsed by the publisher.
